# Repair of Segmental Bone Defect Using Totally Vitalized Tissue Engineered Bone Graft by a Combined Perfusion Seeding and Culture System

**DOI:** 10.1371/journal.pone.0094276

**Published:** 2014-04-11

**Authors:** Lin Wang, Xiang-Yu Ma, Yang Zhang, Ya-Fei Feng, Xiang Li, Yun-Yu Hu, Zhen Wang, Zhen-Sheng Ma, Wei Lei

**Affiliations:** 1 Department of Orthopedics, Xijing Hospital, Fourth Military Medical University, Xi'an, Shaanxi, People's Republic of China; 2 School of Mechanical Engineering, Shanghai Jiao Tong University, State Key Laboratory of Mechanical System and Vibration, Shanghai, People's Republic of China; University of Rochester, United States of America

## Abstract

**Background:**

The basic strategy to construct tissue engineered bone graft (TEBG) is to combine osteoblastic cells with three dimensional (3D) scaffold. Based on this strategy, we proposed the “Totally Vitalized TEBG” (TV-TEBG) which was characterized by abundant and homogenously distributed cells with enhanced cell proliferation and differentiation and further investigated its biological performance in repairing segmental bone defect.

**Methods:**

In this study, we constructed the TV-TEBG with the combination of customized flow perfusion seeding/culture system and β-tricalcium phosphate (β-TCP) scaffold fabricated by Rapid Prototyping (RP) technique. We systemically compared three kinds of TEBG constructed by perfusion seeding and perfusion culture (PSPC) method, static seeding and perfusion culture (SSPC) method, and static seeding and static culture (SSSC) method for their *in vitro* performance and bone defect healing efficacy with a rabbit model.

**Results:**

Our study has demonstrated that TEBG constructed by PSPC method exhibited better biological properties with higher daily D-glucose consumption, increased cell proliferation and differentiation, and better cell distribution, indicating the successful construction of TV-TEBG. After implanted into rabbit radius defects for 12 weeks, PSPC group exerted higher X-ray score close to autograft, much greater mechanical property evidenced by the biomechanical testing and significantly higher new bone formation as shown by histological analysis compared with the other two groups, and eventually obtained favorable healing efficacy of the segmental bone defect that was the closest to autograft transplantation.

**Conclusion:**

This study demonstrated the feasibility of TV-TEBG construction with combination of perfusion seeding, perfusion culture and RP technique which exerted excellent biological properties. The application of TV-TEBG may become a preferred candidate for segmental bone defect repair in orthopedic and maxillofacial fields.

## Introduction

Tissue engineered bone graft (TEBG), with integration of three dimensional (3D) scaffold and osteoblastic cells, may promote bone healing without the undesirable side effects associated with autograft and allograft [1]–[3]. It is based on the hypothesis that osteoblastic cells, either implanted or further recruited to the bone defects, can promote rapid bone regeneration and reunion [4]. For this reason, we propose the “Totally Vitalized TEBG” (TV-TEBG) which is characterized by abundant and homogenously distributed osteoblastic cells with enhanced cell proliferation and differentiation. It is considered to be an objective for bone tissue engineering which maximizes the use of existing construction strategies.

The *in vitro* cell seeding and culture conditions are the most critical factors to TEBG vitalization. Cell seeding, as the first step, crucially determines the following progression of TEBG formation [5]. Although static cell seeding methods are by far the most commonly used approaches [6], [7], perfusion seeding technique provides a higher initial seeding density and more uniform cell distribution even within large scaffolds [8]. Likewise, flow perfusion culture uses a pump to perfuse culture medium directly through the porous scaffold. Not only does it mitigate nutrient transport limitation but also provides mechanical stimulation to seeded cells in form of fluid shear stress. Accordingly, compared with static culture, it improves homogeneous cell distribution and osteogenic differentiation [9]–[12].

In addition to seeding and culture conditions, the scaffold architecture is another important factor affecting the TEBG vitalization. The structure features including porosity, pore size, pore morphology and interconnectivity are widely recognized as important parameters for cell seeding, migration, growth and new tissue formation in three dimensions [13]–[16]. Some previous studies even indicated that cellular behavior and de novo tissue modeling could be affected by scaffold structural features [17], [18]. However, traditional fabricating methods take minimal control of scaffold internal architecture [19], which always lead to unpredictable and nonuniform fluid flow configuration and cell feeding [20]. Rapid Prototyping (RP) technique, also known as solid free form fabrication, allows the fabrication of porous scaffolds with a well-defined architecture on the basis of virtual 3D model data [21]. The RP scaffold allows for the uniform flow distribution with optimized mass transport of nutrients [22], thus supports homogeneous cell migration, proliferation and differentiation [23], [24].

There have been numerous researches investigating the above key points of TEBG construction. F.W. Janssen et al. [25] once designed a direct perfusion bioreactor system capable of seeding and proliferating goat bone MSCs onto clinically relevant BCP ceramic scaffolds successively, but the scaffold structure was not accurately controlled. M. Schumacher et al. [26] and our previous study [27] used ceramic scaffolds fabricated by RP technique combined with perfusion culture method, but the cell seeding condition was still static. Therefore, in the current study, we assume that with the combination of perfusion seeding, perfusion culture and well-defined scaffold architecture fabricated by RP technique, it is possible to construct the TV-TEBG and obtained favorable healing efficacy of the segmental bone defect. So the purpose of the study is (1) to investigate the feasibility of the combined strategy for constructing the TV-TEBG, (2) to explore its healing efficacy on segmental bone defect.

## Materials and Methods

### Scaffold fabrication

**Figure 1:**
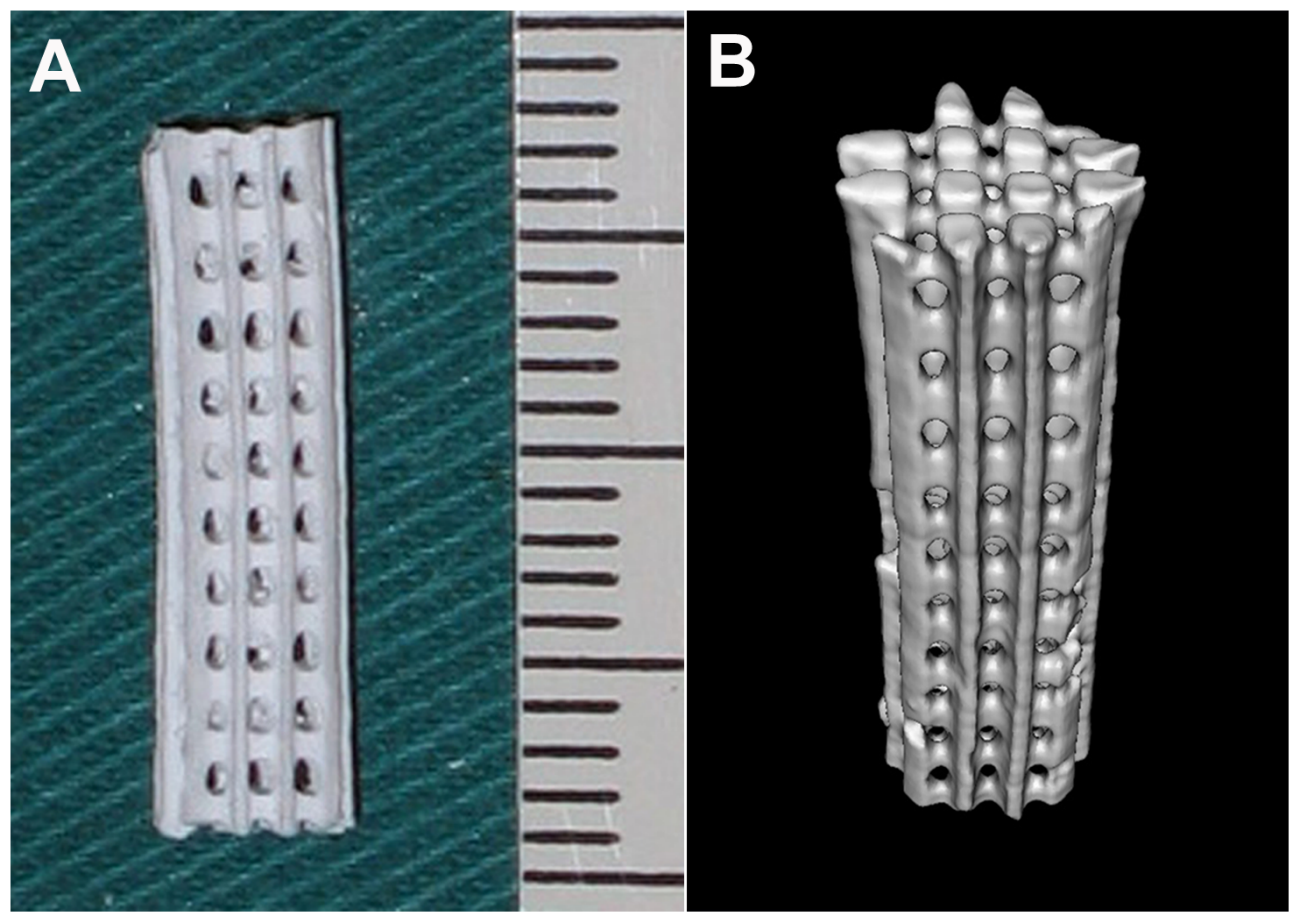
The gross view (A) and micro-CT scan (B) of β-TCP scaffold fabricated by Rapid Prototyping technique with designed internal architecture.

β-TCP scaffolds were fabricated with a process including the scaffold design using computer-aided design (CAD) software, β-TCP (β-Ca_3_(PO_4_)_2_) suspension, and epoxy molds manufacture by SL [28]. Commercial β-TCP powder obtained from Edward Keller (Shanghai, China) had an average particle size of 2.3 µm with a surface area of 1–6 m^2^/g. Prepared β-TCP suspension was cast into the molds and sintered at 1100°C, so that the epoxy mold with interconnected beams could be removed by pyrolysis to create the channel space in the scaffolds. The scaffold with 4 mm in diameter and 16 mm in length had a homogeneous porosity of 42% (Fig. 1A). The internal architecture contained channels separated at equal distance, which were allowed to penetrate through the scaffold orthogonally in X, Y, and Z direction to mimic a simple 3D interconnected structure. Both the inner diameter of the channel and the width of the solid wall between the channels were 300 µm. The morphology and 3D structure of the scaffold were characterized by micro-computerized tomography (micro-CT, eXplore LocusSP, GE Healthcare, Canada) at a resolution of 40 µm (Fig. 1B). The scaffolds were sterilized by Co60-γ radiation before use.

### Flow perfusion seeding and culture system

**Figure 2:**
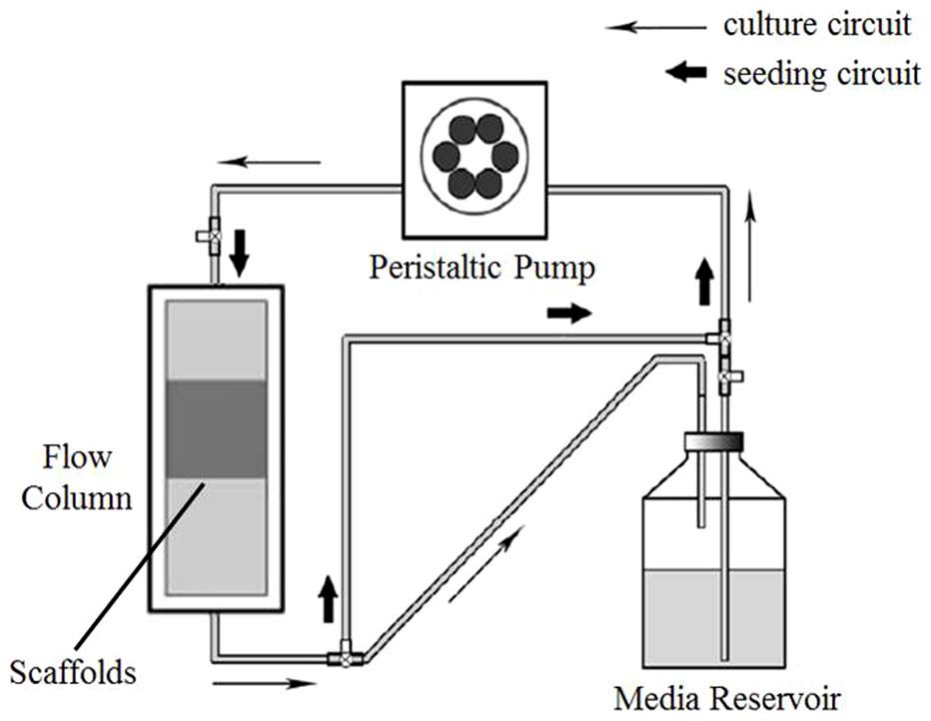
Flow circuit schematic diagram in the flow perfusion seeding and culture bioreactor. The scaffolds were press-fit into the column and the medium was pumped into the scaffolds via a peristaltic pump. The arrows indicated the flow direction of the seeding and culture medium.

A flow perfusion seeding and culture system was developed (Fig. 2). The system consisted of an eight-channel peristaltic pump (ISMATEC IP65, Switzerland) and eight independent pumping circuits with separated flow columns and reservoirs. Each circuit was a double-circuit structure which consisted of flow column, media reservoir, tubes and three-way stopcock. The seeding and culture circuits could be switched by adjusting the direction of the stopcock. In this study, cell suspension and culture medium were pumped continuously at a controllable flow rate through the cell/scaffold composites along the seeding and culture circuits respectively.

### Cell culture

Osteoblasts were isolated and extracted from calvaria of 1-day-old New Zealand White rabbits. The animal experiments were performed in accordance with the protocols approved by the Institutional Animal Care Committee of Xijing Hospital. Primary osteoblast culture was established using an enzymatic isolation method [29]. Briefly, the calvaria tissues were extracted and rinsed with phosphate-buffered saline (PBS) (containing penicillin and streptomycin). Afterwards, bone fragments were minced and digested with trypsin for 15 min at 37°C. After an intensive washing in PBS, bone fragments were transferred to culture flask, where cell outgrowth was observed within 3–5 days under normal culture conditions: Dulbecco's modified Eagle's medium (DMEM; Gibco) supplemented with 10% fetal calf serum (FCS; Gibco), 50 µg/mL gentamicin, 100 µg/mL ampicillin, 2.5 µg/mL fungizone. The cells were cultured at 37°C in a humidified atmosphere of 5% CO_2_ and maintained in culture by passaging once per week. Culture medium was changed twice per week. Cells in the third passage were routinely trypsinized, counted, and seeded onto the scaffolds for all the experiments.

### Cell seeding and cell/scaffold composites culture

Scaffolds were placed in 12-well plates, washed with complete culture medium, immersed in FCS and left in an incubator overnight to pre-wet the scaffold surface. Then the medium was aspirated and all the scaffolds were divided randomly into 3 groups: the perfusion seeding and perfusion culture (PSPC) group, the static seeding and perfusion culture (SSPC) group, and the static seeding and static culture (SSSC) group.

PSPC group: The scaffold cluster (including six scaffolds) were put into the flow columns directly. A cell suspension of 6×10^6^ cells in 10 mL culture medium were then added into every seeding circuit with the flow rate of 1 mL/min for 24 h in a standard cell culture incubator with the environment of 37°C and 5% CO2. Afterwards, the seeding circuits were closed and the culture circuits with each reservoir filled with 50 mL osteogenic medium [DMEM supplement with 50 µg/mL L-ascorbic acid, 10 mmol/L β-glycerophosphate, and 10 nmol/L dexamethasone] were opened. The culture flow rate was initially set at 0.5 mL/min for 1 day to allow better cell attachment before being increased to 2 mL/min for the duration of culture.

SSPC group: In order to keep the same amount of seeded cells with PSPC group, a cell suspension of 6×10^6^ cells in 2 ml were dripped onto the scaffold cluster and incubated for 2 h. Then culture medium was added to cover the scaffolds. Cells were allowed to attach overnight, then all the scaffolds were placed into the flow column respectively. The dynamic culture procedure was the same as the PSPC group.

SSSC group: After cellular attachment overnight as mentioned above, the composites were placed into new 12-well plates respectively and osteogenic medium was added to the wells and changed twice per week.

All the three groups were cultured *in vitro* for 8 days.

### Glucose consumption

The cellular consumption of D-glucose was determined via spectrometry according to protocol provided by Roche (D-glucose UV-method, Roche, Germany). In brief, at each medium change, the samples were collected and diluted appropriately to stay within the detection range of the assay and measured three times to obtain the average value. The assay was performed following the instructions offered by kit manufacturer, and the absorbance of each cuvette at 340 nm was measured with a spectrophotometer. The fresh complete medium and the D-glucose control solution were also processed and served as the reference. Finally, the glucose concentration was quantified based on the absorbance difference and the glucose consumption was expressed as the average daily glucose reduction in the medium.

### Cell viability

Cell viability on the scaffolds was assessed by MTT (3-(4,5-dimethylthiazol-2-yl) -2,5-diphenyltetrazolium bromide) assay at the end time point of culture. At the 8th day, the composites from each group were randomly selected, transferred to individual well of 12-well plates and cut into approximate 2–3 mm^3^ fragments. MTT solution was added and the composites were incubated at 37°C for 4 h to form formazen, which was then dissolved using dimethyl sulfoxide (DMSO). The optical density (OD) was measured using a spectrophotometer at 490 nm. All the fragments were collected, dried at 56°C overnight and weighted. At least three samples were analyzed for each condition and the cell viability was expressed as the absorbance per gram of scaffold.

### Alkaline phosphatase (ALP) activity

The ALP activity of each sample was measured at 2, 4, 6 and 8 days of culturing respectively. Briefly, the constructs were cut into 2–3 mm^3^ fragments and sonicated. Followed by vortex and centrifugation, the supernatant was collected and diluted as necessary. The ALP activity was determined by a colorimetric assay using an ALP reagent containing p-nitro-phenyl phosphate (p-NPP) as the substrate. The absorbance of p-nitrophenol formed was measured at a wavelength of 405 nm. All samples were run in triplicate. Protein concentration was estimated by using the protein assay kit (Pierce, Rockford, IL). All the fragments were dried at 56°C overnight and weighted. The ALP activity was expressed as μmol/h/mg of protein/g of scaffold.

### Histological and Histomorphometric analysis

Three cell/scaffold composites selected from each group at 8th day of culture were fixed in 10% formalin solution for 7 days. After fixation, the samples were dehydrated in a gradient ethanol series up to 100%, cleared with toluene, and embedded in methylmethacrylate. After polymerization, the serial sections about 50 µm thick were performed on a Leica cutting and grinding system (Wetzlar, Germany) along longitudinal axis. Then the sections were stained with Haematoxylin and Eosin (HE) staining and examined with a standard light microscope (Leica LA Microsystems, Bensheim, Germany) equipped with a digital image capture system (Penguin 600CL, Pixera). The stained sections were observed for status of cell amount and distribution within the scaffold. The cell area was quantitatively measured with the aid of an image analysis system (Image-Pro Plus software, Media Cybernetics, Silver Spring, USA).

### Animals ethics and animals grouping

All animal experiments were performed in accordance with the National Institutes of Health Guidelines for the Use of Laboratory Animals. The animals used in the current study were obtained from protocols approved by the Fourth Military Medical University Committee on Animal Care. Critical-sized segmental defects of rabbit radius were used to evaluate the defect healing efficacy of different kinds of cell/scaffold composites. Eighteen male New Zealand white rabbits which were 3–4 months old with body weight of 2.0–2.5 kg were used. A total of 36 radius defects were randomly divided into 6 groups with 8 defects in PSPC, SSPC, SSSC groups respectively, and 4 defects each in pure β-TCP scaffold, autograft and untreated group (Table 1). The composites of the three experimental groups used for transplantation were treated the same as those for the *in vitro* study.

**Table 1:** The implants performed in each experimental group.

Groups	Number of tested samples
PSPC	8
SSSC	8
SSSC	8
Pure β-TCP scaffold	4
Autograft	4
Untreated	4

### Surgical procedures

Rabbits were anesthetized with an intramuscular injection of 50 mg/kg ketamine hydrochloride and 5 mg/kg xylazine. The segmental defect models were created as previously described [30]. Briefly, bilateral 16-mm segmental bone defects were created in the middle of the radius diaphysis. The defects were irrigated with sterile physiological saline solution and the composites were implanted into the defects. Then muscles, fascia and skin were separately closed over the defects. After the operation, all the rabbits were allowed to move freely in their cages without external support and received intramuscular injection of gentamycin as an antibiotic prophylaxis.

### Radiological examination

The rabbits were lightly anesthetized at 1 day and 12 weeks postoperatively. Then the standardized radiographs of the bilateral forearms were obtained to observe the bone healing. The X-ray films were graded according to the scoring system reported by Yang' s group (Table 2) at 12 weeks [31]. The scale was composed of four categories: (1) The first category evaluated periosteal reaction graded from 0 to 4 points. (2) The second category evaluated osteotomy line graded from 0 to 4 points. (3) The third category evaluated remodeling graded from 0 to 2 points. (4) The fourth category evaluated graft appearance graded from 0 to 4 points. A graft that had fully consolidated and completely reorganized would receive a maximum total score of 42 points. The X-ray images were scored and compared among experimental groups at 12 weeks postoperatively.

**Table 2:** Radiographic grading system.

Grading items	Score
Periosteal Reaction*	
None	0
Minimal [localized to the gap]	1
Medium [extends over the gap; <1/4]	2
Moderate [< 1/2 but >3/4]	3
Full	4
Osteotomy Site*	
Osteotomy line completely radiolucent	0
Osteotomy line partially radiolucent	2
Osteotomy line invisible	4
Remodeling*	
None apparent	0
Intramedullary space	1
Intracortical	2
Graft Appearance*	
Unchanged/intact	0
Mild resorption	1
Moderate replacement	2
Mostly replaced	3
Fully reorganized	4

*Proximal, distal, and central part of graft scored individually.

### Fluorescence labeling

Sequential fluorochrome markers were administered to monitor the mineralization process of new bone formation, all the rabbits were injected intramuscularly with Tetracycline (40 mg/kg, Sigma, USA) 2 weeks and 3 days prior to euthanasia. After X-ray films were taken at the end of 12 weeks, all rabbits were sacrificed by an overdose of pentobarbital. Four bone specimens at the defect regions from each experimental group (PSPC, SSPC and SSSC) were harvested for fluorescence analysis.

### Biomechanical testing

Immediately after the animals were sacrificed, the specimens were extracted from sacrificed rabbits (n = 4 in each group). The mechanical properties were assessed using a destructive compression test. Specimens were sawed to cubes of 8 mm×4 mm×4 mm and wrapped in PBS-soaked gauze to maintain moisture during preparation process until testing. The compressive load with a cross-head speed of 1 mm/min was applied using the testing machine (AGS-10kN, Kyoto, Japan) until fracture occurred. The compressive strengths were calculated by the values of geometric area and compressive load of the specimens from the load-displacement curve.

### Histological analysis

The other retrieved specimens were placed in formalin solution for 2 weeks (n = 4 in each group). Following fixation in graded ethanol (80–100%), cleared with toluene, all the specimens were embedded in methylmethacrylate. After polymerization, the serial sections about 50 µm thick were performed on a Leica cutting and grinding system (Wetzlar, Germany) along longitudinal axis. The labelled sections were observed under fluorescence microscopy (Penguin 600CL, Pixera) before stained. Then the sections were stained with 1.2% trinitrophenol and 1% acid fuchsin (Van Gieson staining) and examined with a standard light microscope (Leica LA Microsystems, Bensheim, Germany) equipped with a digital image capture system (Penguin 600CL, Pixera).

### Histomorphometric study

Bone and material were pseudocoloured respectively using Adobe Photoshop 6.0 prior to histomorphometric analysis, and measured using an image analysis system (Image-Pro Plus software, Media Cybernetics, Silver Spring, USA) then. Bone formation was quantified from the pixels that represented bone tissue, while the total area was defined as the implant section. The rate of new bone formation was presented as the percentage of bone area in total implant area [(bone area/total area) ×100%]. Moreover, the mineralization apposition rate (MAR, the vertical interval between parallel fluorochrome markers/days of implantation) was analyzed from the images of fluorochrome labeling.

### Statistical analysis

Results were expressed as means ± standard deviation. Statistical analysis was performed using a one-way analysis of variance (ANOVA) followed by a Student-Newman-Keuls test at a 95% confidence interval.

## Results

### Glucose consumption

**Figure 3:**
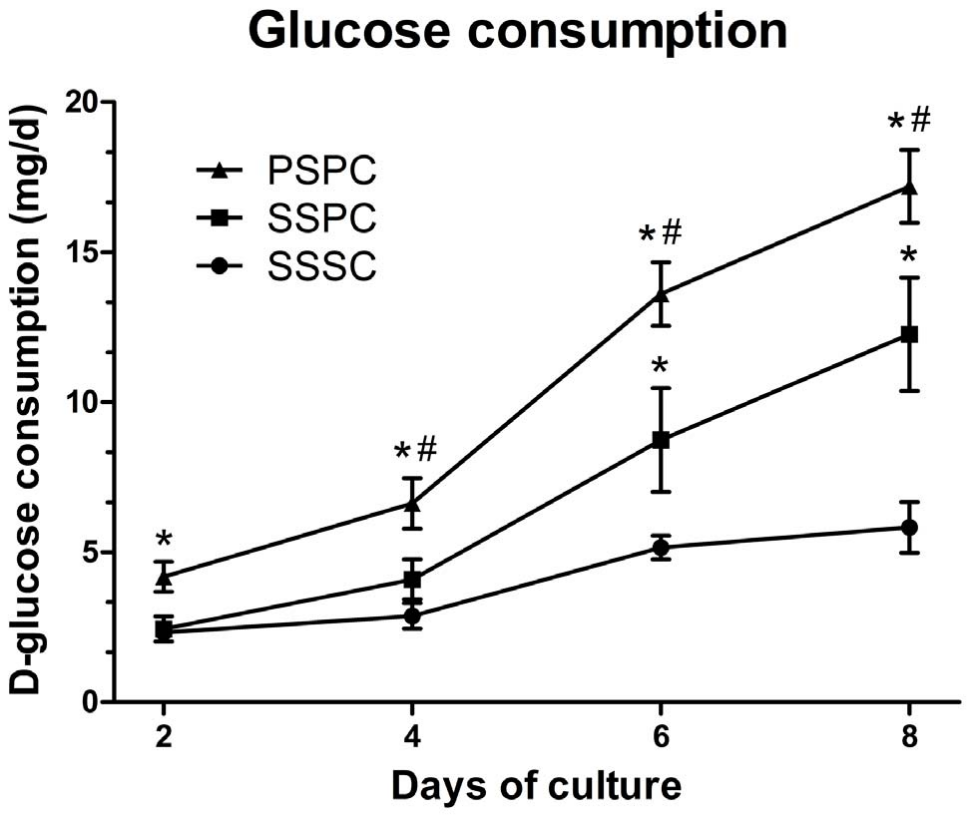
Analysis of the D-glucose consumption of the cell/scaffold composites at different time points. PSPC group exhibited higher D-glucose consumption at all time points followed by SSPC group. ^*^ *p*<0.05 vs. SSSC; ^#^ *p*<0.05 vs. SSPC.

Glucose consumption of composites cultured under different seeding and culture conditions was measured to study their metabolic activities reflecting cell quantity on the scaffolds. The daily D-glucose consumption rate increased steadily with time in all the groups (Fig. 3). PSPC group exerted significantly higher glucose consumption than the static seeding groups at all the time points, achieving 1.4 and 2.95 folds higher than SSPC and SSSC groups respectively at day 8. Despite of no significant difference at day 2 and 4 between SSPC and SSSC groups (*p*>0.05), SSPC group showed a significantly higher amount of glucose consumption since day 6 (*p*<0.05).

### Cell viability

**Figure 4:**
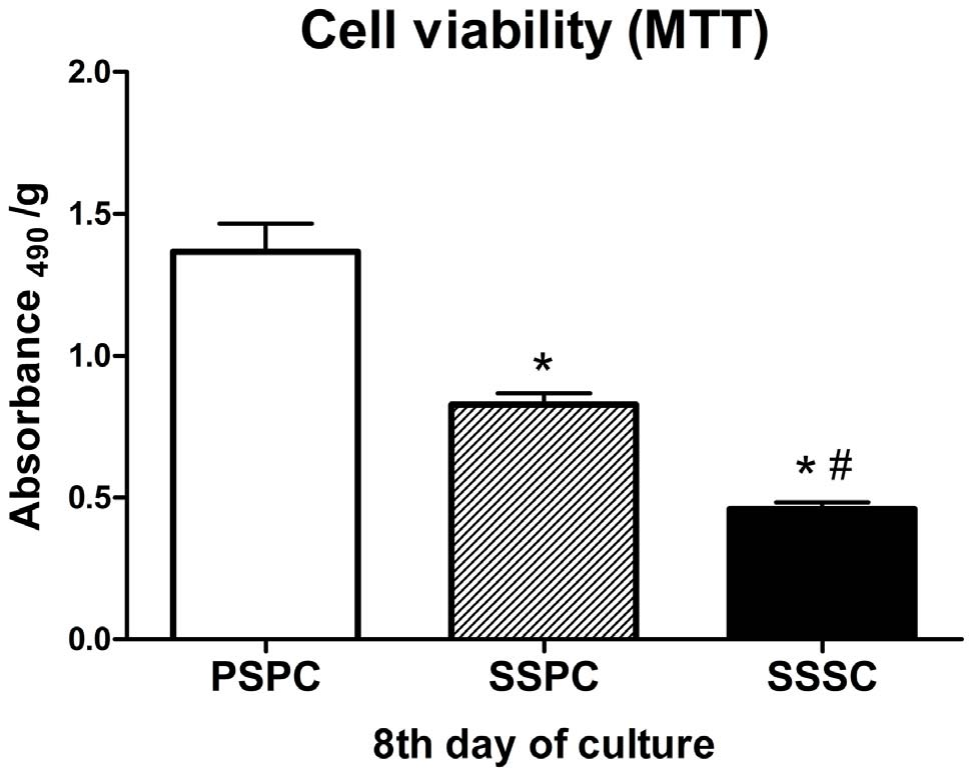
Assessment of cell viability of the cell/scaffold composites at 8th day of culture. PSPC group exhibited higher cell viability followed by SSPC group. ^*^ *p*<0.05 vs. PSPC; ^#^ *p*<0.05 vs. SSPC.

Proliferation of cells on the scaffolds was indicated by the cell viability, which could be determined by MTT colorimetric method. The increase of cell viability with time during the culture was similar to the increase of the daily glucose consumption in all groups. Fig. 4 depicted the cell viability after 8 days cultivation. PSPC group demonstrated the higher cell viability, followed by SSPC group (*p*<0.05).

### ALP activity

**Figure 5:**
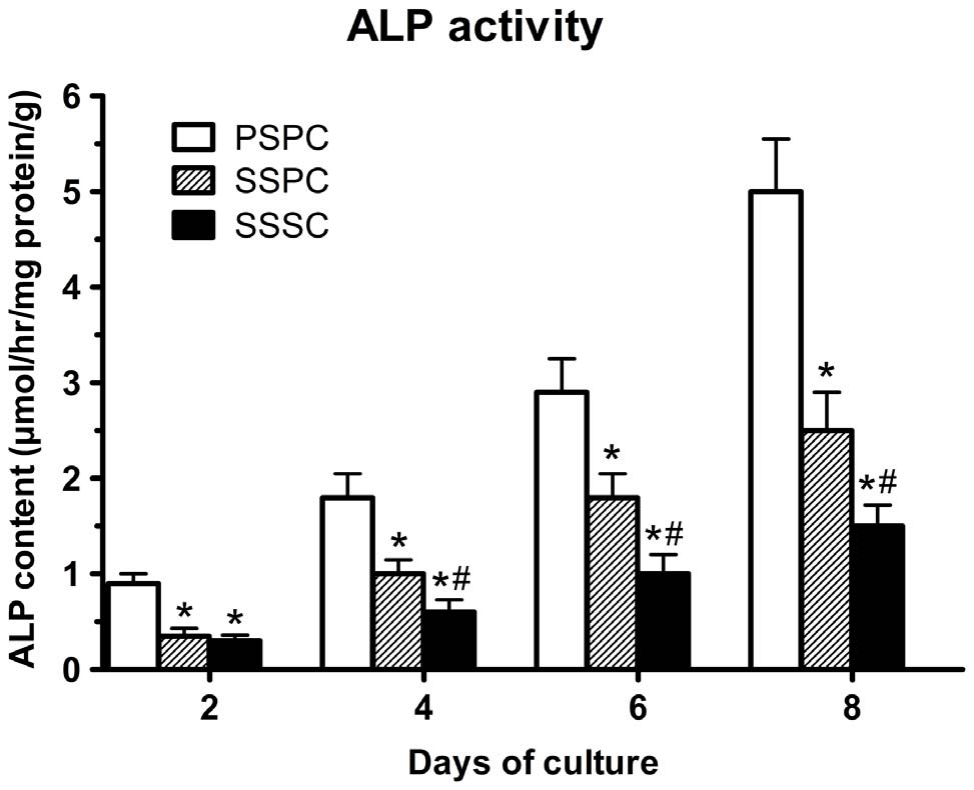
Measurement of ALP activity of cell/scaffold composites at different time points. PSPC group exhibited higher ALP activity followed by SSPC group.^*^ *p*<0.05 vs. PSPC; ^#^ *p*<0.05 vs. SSPC.

Differentiation of cells cultured on the scaffolds at 2, 4, 6 and 8 days of incubation was assessed by ALP activity and illustrated in Fig. 5. The ALP activity increased from day 2 to day 8 among all groups. At day 2, there was no obvious difference between SSSC and SSPC groups (*p*>0.05) but significant difference between SSPC and PSPC group (*p*<0.05). For the following culture period, PSPC group continuously displayed higher differentiation capacity followed by SSPC group (*p*<0.05).

### Histological and Histomorphometric study

**Figure 6:**
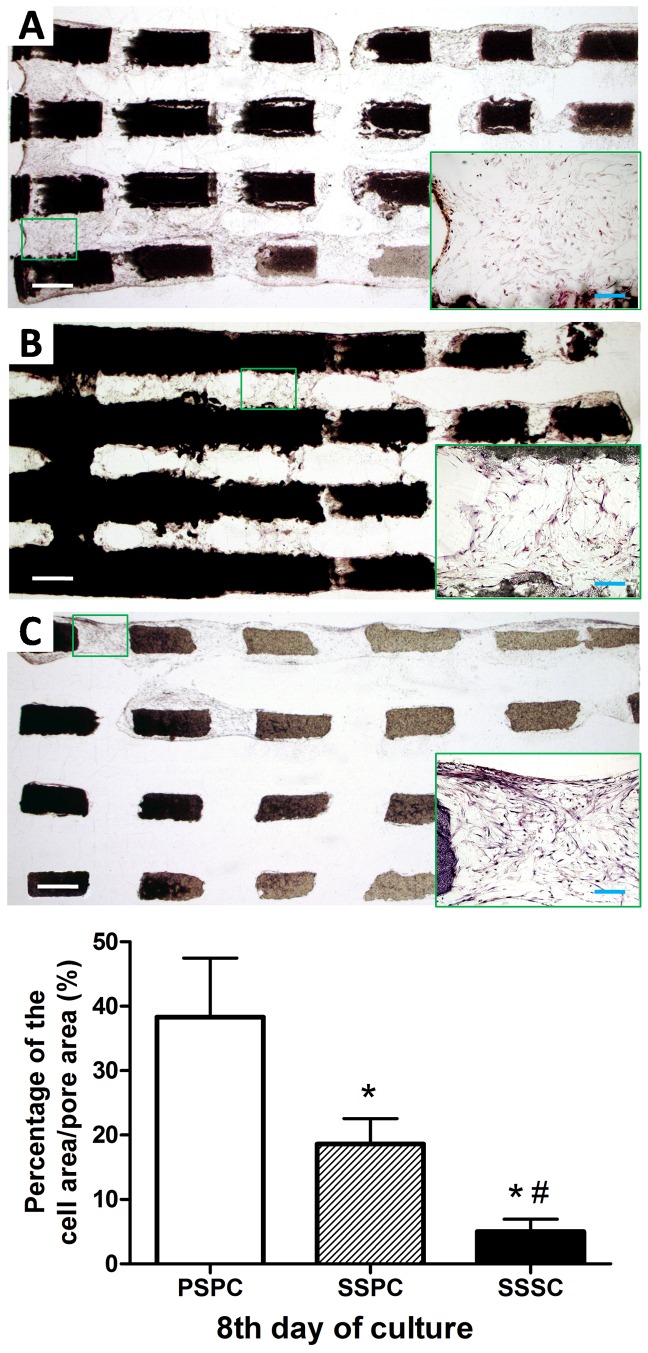
Histological analysis of the cell/scaffold composites at 8th day of culture. The cells (blue) and mineralized matrix (red) were visualized by hematoxylin and eosin (H&E) staining. A: PSPC, B: SSPC, C: SSSC. Scale bar: 50 µm (white), 20 µm (blue). Quantitative analysis indicated the higher percentage of cell area/pore area of PSPC group followed by SSPC group. ^*^ *p*<0.05 vs. PSPC; ^#^ *p*<0.05 vs. SSPC.

The stained sections of cell/scaffold composites were shown in Fig. 6. After 8 days culture *in vitro*, PSPC and SSPC groups demonstrated layers of cells and extracellular matrix on the scaffold surface as well as inner wall of the channels. In PSPC group, many channels were almost filled with multilayer cells and red-stained mineralized matrix. In SSPC group, cell layers attached on the inner wall of channels were observed to band together, but the area of cell occupation was smaller than that in PSPC group (*p*<0.05). In SSSC group, cells appeared only on the outer surface of the scaffold and were almost absent in the central part. The percentage of the cell area/pore area in PSPC, SSPC and SSSC group was 38.3±6.5%, 18.61±2.79% and 5.02±1.35% respectively. Statistical significance was found between either two of the three groups (*p*<0.05).

### Radiographic examination

**Figure 7:**
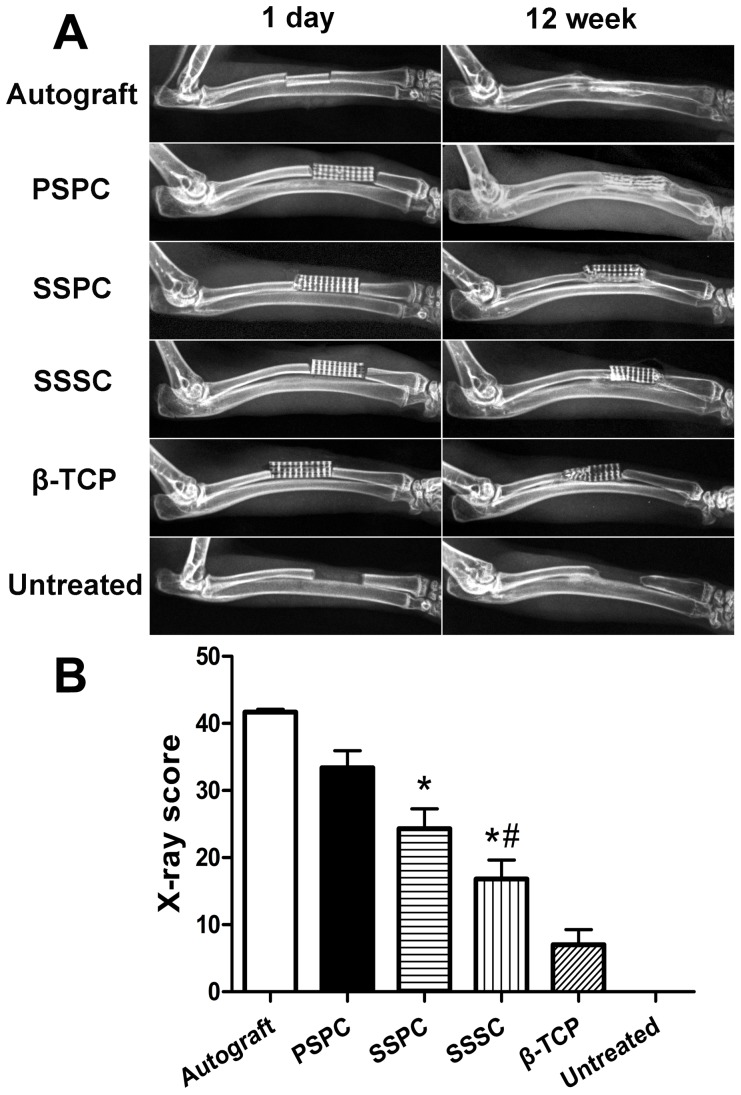
The radiographic examination of radius segmental defects repaired with different grafts. (A) The radiographic images of all the groups at 1 day and 12 weeks. (B) Quantitative evaluation indicated the highest score of PSPC group except for autograft, followed by SSPC group at 12 weeks. ^*^ *p*<0.05 vs. PSPC; ^#^ *p*<0.05 vs. SSPC.

The X-ray images at 1 day and 12 weeks postoperatively were shown in Fig. 7. The high-density radiopaque areas of TEBG were clearly identified at bone defects that indicated they were in accurate position after 1 day postoperatively. At 12 weeks, there was massive bony connection between the implants and bone tissue as a shell-like radiopacity of new bone became noticeable in PSPC group. A part of the implanted material was degraded and the boundary between newly formed and normal bone disappeared, which demonstrated the bone defect was well repaired. In SSPC group, a sand-like radiopacity was observed and implants became fuzzy at the ends of the defect, which indicated a small amount of callus had formed in the interspaces between the implants and the host bone tissue. Only a little bony connection appeared in SSSC group between the implanted material and the host bone tissue. The radiolucent areas remained much larger than PSPC and SSPC groups. In β-TCP group, radiolucent boundary line at the cutting ends was still clear indicating the incomplete repair. While in autograft, the bone defect was fully repaired. The X-ray scores in each group were 41.2±0.8, 33.4±1.8, 24.3±2.1, 16.8±2.0, 7.0±1.6 and 0 for autograft, PSPC group, SSPC group, SSSC group, β-TCP group and untreated group respectively. There were statistical differences between any two groups (*p*<0.05).

### Biomechanical Testing

**Figure 8:**
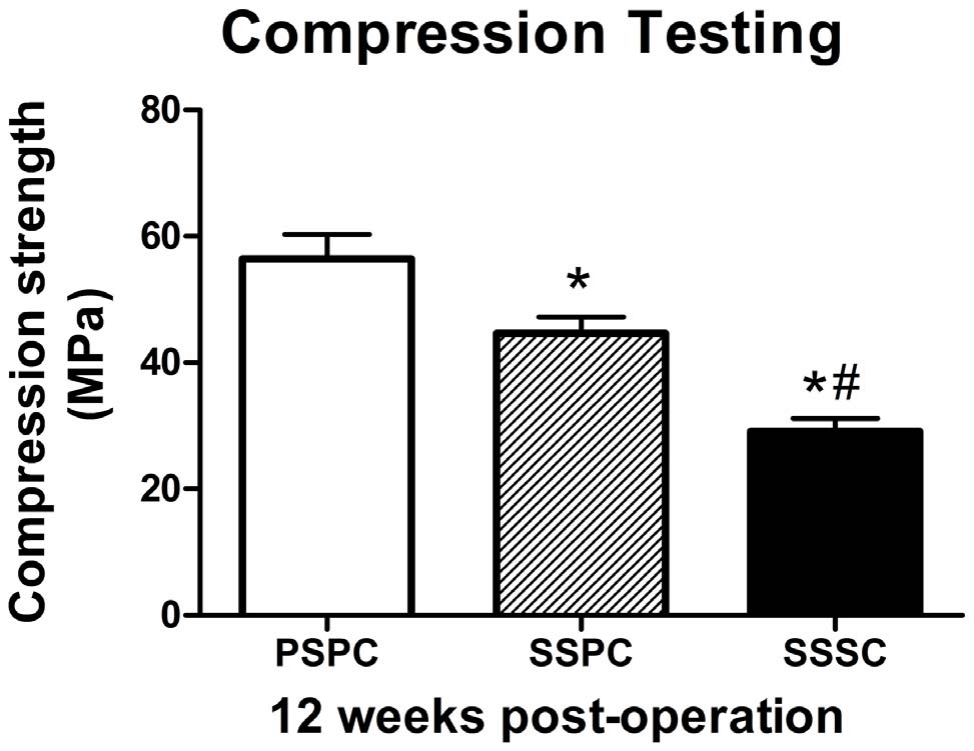
Compression testing results at 12 weeks post-operation. PSPC group exhibited higher compression strength followed by SSPC group. ^*^ *p*<0.05 vs. PSPC; ^#^ *p*<0.05 vs. SSPC.

To evaluate the functional/mechanical properties of the new bone regeneration and its integration within host tissue, we carried out compression tests on the extracted specimens at 12 weeks post-operation (Fig. 8). The PSPC group showed the highest compression strength (54.46±3.84 MPa). SSPC group demonstrated significantly higher compression strength (44.63±2.62 MPa) than that of SSSC group (29.13±2.01 MPa, *p*<0.05) but lower than PSPC group (*p*<0.05). The compression strength of autograft was 70±3.6 Mpa in the present studies (data not shown in the figure).

### Fluorescence Labeling

**Figure 9:**
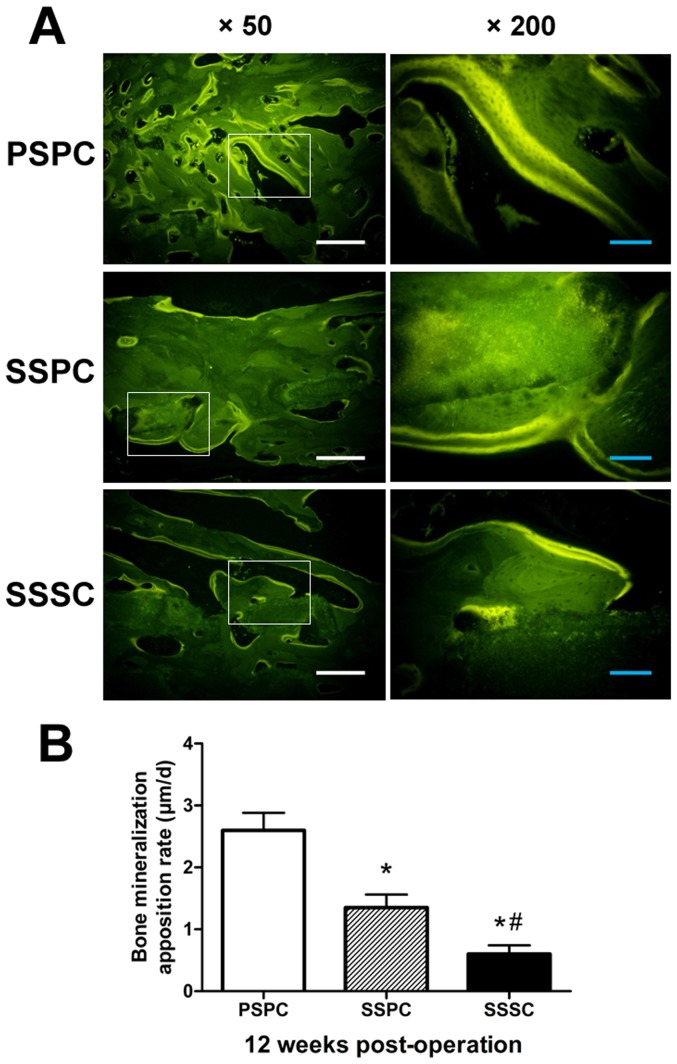
Fluorochrome labeling of bone regeneration at 12 weeks post-operation. (A) The fluorescent labeling images of extracted specimens at 12 weeks post-operation. Scale bar: 50 µm (white), 10 µm (blue). (B) Quantitative analysis indicated the faster mineralization apposition rate of PSPC group followed by SSPC group. ^*^ *p*<0.05 vs. PSPC; ^#^ *p*<0.05 vs. SSPC.

Fluorescence labeling was detected in all the residual specimens. Fluorescence images of the sequential fluorochrome labels revealed the dynamic process of new bone formation in the scaffolds (Fig. 9A). The quantitative analysis of fluorochrome markers interval showed that the MAR of PSPC group was significantly higher than that of SSPC and SSSC groups (*p*<0.05), followed by SSPC group whose MAR was higher than SSSC group (*p*<0.05, Fig. 9B).

### Histological assessment

**Figure 10:**
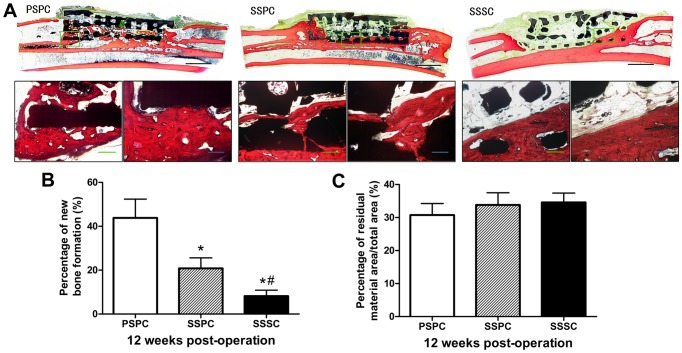
Histological analysis of regenerated bone at 12 weeks post-operation. (A) The newly formed bone was stained in red color with visible cell nuclei by Van Gieson staining at 12 weeks post-operation. PSPC group exhibited isolated new bone islands (green arrow) in the center of the scaffold. Scale bar: 5 mm (black), 20 µm (green), 10 µm (blue). (B) Histomorphometric analysis showed that the percentage of new bone formation in PSPC group was significantly higher than that of SSPC group, followed by SSSC group. ^*^ *p*<0.05 vs. PSPC; ^#^ *p*<0.05 vs. SSPC. (C) The percentage of residual material area/total area. There were no statistical differences among the three groups (*p*>0.05).

To evaluate the tissue response to the implanted composites and the defect healing progress, histological analysis was performed on the tissue/biomaterial interface and the area of the implant. As shown in Fig. 10, there was much more substantial and relatively more uniform new bone formation throughout the implant in PSPC group than those in other groups. The islands of newly formed bone appeared independently distributed or connected and even formed woven bone structures mostly close to ulnar. In SSPC group, new bone formation was detected both on the surface and periphery of internal channel of scaffolds close to the ulna but less than PSPC group. In SSSC group, the scaffolds were partly filled with a small amount of new bone in the periphery. The percentage of new bone formation (bone area/total area) in PSPC group was 43.84±8.53%, which was significantly higher than 20.84±4.78% of SSPC group (*p*<0.05), followed by 8.17±2.73% of SSSC group (*p*<0.05). The percentage of residual material area/total area in PSPC group was 30.76%±3.5%, lower than that of SSPC (33.81±3.69%) and SSSC (34.58%±2.84%), but the differences were not significant among the three groups (*p*>0.05).

## Discussion

Taking advantages of the custom designed perfusion seeding and perfusion culture (PSPC) system combined with well-defined β-TCP scaffold fabricated by RP technique, we successfully constructed the TV-TEBG loaded with abundant and homogenously distributed osteoblasts with enhanced cell proliferation and differentiation. Its biological performance in repairing segmental bone defect was much better than SSPC and SSSC groups in radiographic examination, biomechanical testing and histological assessment, closest but was still not comparable to the performance of autograft.

Optimal TEBG construction process should combine high cell seeding efficiency with preferential culture condition to maximize cell utilization in order to meet the vitalization target. F.W. Janssen *et al*. [25] once designed a direct perfusion bioreactor system capable of seeding and proliferating goat bone MSCs onto clinically relevant BCP ceramic scaffolds successively. Zhao F [32] also used combined perfusion seeding and perfusion culture method for long-term development of 3D engineered tissue with human MSCs. Compared with spinner flask or rotating wall vessel bioreactor, perfusion bioreactor has been demonstrated to enhance the induction and osteogenesis of the murine bone marrow stromal cells and result in better cell uniformity and cell functions [33]. In the present experiment, with combination of well-defined RP scaffold, we designed a new kind of flow perfusion bioreactor in which the full process of tissue regeneration, from cell seeding to cell culture, could be performed in consecutive steps. As a result, we achieved continuous tissue reconstruction which obtained much better results of cell growth, distribution and differentiation throughout the entire scaffold.

It is demonstrated that during the stage of the perfusion seeding, the dynamic flow affords more energetically favorable places and the stronger cell-matrix interaction is developed with the continuous flow stimulation in the following stage of perfusion culture [34]. In addition, through active adhesion to the scaffold, perfusion seeding restricts cell deposition to viable cells whereas cells are passively embedded on the scaffold in static seeding—irrespective of their viability [35]. In SSPC group, the unvital osteoblasts have little response to the flow stimulation and may impede the proliferation and differentiation of vital ones by occupying the limited space and supply of nutrition and oxygen within the scaffold, as evidenced by lower glucose consumption and ALP activity than those of PSPC group at 2 days of culture. Furthermore, the mix of unvital cells makes cellular clusters be washed away much easier when subjected to shear forces in the flow perfusion culture. Last but not the least, the PSPC system is advantageous to maximize cell utilization, to minimize the disturbance of TEBG construction which maintains the structural and functional integrity of the TEBG, and to reduce the complexity of operation as well as the risk of contamination in one single closed bioreactor system [32].

The structural property of the scaffold plays another important role in TEBG construction [18]. RP scaffold provides regular frameworks so that seeded cells orient and adhere along channels uniformly in initial stage [24]. Under flow perfusion condition, the scaffold architecture preferentially orients the flow in a specific direction, determines interconnection and accessibility of the fluid flow [36] that contributes to the creation of a regular 3D flow, and eventually improves cell proliferation and differentiation than static condition [24]. Seeded or proliferated cell clusters may easily block fluid flow in random porous microstructure. By contrast, well-defined architecture combined with flow perfusion culture maintains an ideal means of transporting nutrients and oxygen flow to the cells by forcing its uniform penetration into the scaffold bulk, thus making homogenous feeding possible [24] [37].

The culture media flow is known to influence osteogenesis through mechanical stimulation of the seeded osteoblastic cells [38]. At the microscopic level, the distribution of shear stress induced by fluid perfusion is much dependent on architecture feature of the scaffold. Previous study demonstrated that the random architecture of the scaffold lead to highly variable shear stresses because of non-uniform fluid flow, contained in the scaffold [22]. Well-defined 3D structure creates preferential paths for culture medium flow thus keeps constant, uniform and relatively low shear stress [37], which imitates the physiological mechanical stimuli of the target tissue *in vivo* [39], [40] and contributes to homogeneous cell proliferation and distribution [24].

The osteogenesis and osteoinduction are mainly dependent on the osteoblastic cells of TEBG. So it is generally considered that cell quantity and viability are key factors to the bone healing efficacy of TEBG [41]. F. Pieri *et al.* [42] demonstrated a dose–response relationship between concentration of adipose stem cells (ASCs) and new bone formation. As the concentration of ASCs increased, the overall mean bone volume and bone apposition to the implant surface increased. Z. Ge *et al.* [4] implanted scaffolds loaded with MSC induced osteoblasts which were transfected by green fluorescent protein (GFP) into the rabbit femur to study the cellular performance. The result showed that after implantation, the osteogenic cells did not only survive, migrate and continually proliferate in the scaffolds, but also secreted growth factors to recruit host cells to migrate into the augmented site and recruited surrounding tissue to grow in. In accordance with previous studies, the TV-TEBG constructed by PSPC method in our study which was proved to yield the highest cell quantity, viability, differentiation capacity also exerted best healing efficacy of segmental bone defect. In addition, we found that cell quantity not only affected the amount of newly formed bone tissue, but also strongly influenced MAR. The width of yellow fluorescent bends traced by tetracycline clearly indicated the fastest MAR in PSPC group. Nair *et al.* [43] once observed the faster bone regeneration, remodeling and material degradation in HASi scaffold seeded with goat MSCs than pure scaffold, in line with our discovery.

Some studies demonstrated that cell distribution within the TEBG directly influenced and even decided the distribution and growth pattern of bone regeneration [44], [45]. Well-defined 3D scaffold loaded with homogeneous osteoblasts and extracellular matrix promote discontinuous ingrowth with separated bone islands throughout the whole scaffold. Discontinuous pattern of bone ingrowth has the advantage that they may be able to fill the entire volume of space within the scaffold more quickly than continuous pattern since bone will be forming not only from the margins but also throughout the whole space of the scaffold [45]. As a result, we observed relatively uniform new bone formation in form of isolated bone islands within the scaffold in PSPC group overweighing those in the other groups. The best radiological examination (the score was only lower than autografts) and mechanical testing results may also attribute to the highest quantity and uniformity of new bone formation related to perfusion seeding and culture conditions.

The distinguished healing efficacy of TV-TEBG cannot match the performance with autograft. Vascularization deficiency which negatively affects nutrient delivering and degradation products removing from biodegradable materials is considered as an obstacle in complete repair of large bone defects [46]. Although the TV-TEBG was constructed with uniformly proliferated osteoblasts *in vitro*, the distribution of new bone tissue within the scaffold *in vivo* was not duplicated. It was observed that a majority of new bone located near the extremities of the segmental defects and the ulnas. The reason might be that the above regions were much closer to the bone graft bed, where the capillary vessel network sprouted in the scaffold and increased the initial survival of transplanted cells and the follow-up new bone regeneration. In contrast, the insufficient vascularazition lead to reduced new bone quantity in other regions. Therefore, it is quite crucial to generate a capillary network, which delivers sufficient nutrients to cells seeded onto the scaffolds for improved healing efficacy of the TEBG after implantation, as shown by many other studies [45], [47], [48]. Another obstacle is the failure of material to timely degrade. A balance should be reached between the rate of new bone remodeling and material degradation [49]. In our study, the PSPC group which exhibited the largest amount of new bone regeneration also demonstrated the highest degradation rate of materials. But the residual material was still as high as 30.76±3.5% after transplantation for 12 weeks. It needs to be further investigated whether the relatively low degradation rate of β-TCP material influences the effect of TV-TEBG on segmental bone defect repair. On the other hand, the porous scaffold maintaining initial profile formed a bone/material chimera with new bone tissue at 12 weeks, which might be beneficial to rapid bone reunion.

## Conclusion

In this study, with combination of perfusion seeding, perfusion culture method and RP technique, we successfully constructed the TV-TEBG which exerted excellent biological performance *in vitro* and favorable healing efficacy of the segmental bone defect *in vivo*. The combined strategy as described here is a promising approach towards the clinical TEBG construction. The application of TV-TEBG may become a preferred candidate for segmental especially large scale bone defect repair in orthopedic and maxillofacial fields.
